# On the mechanism of control of the pulse rate

**DOI:** 10.1113/EP093308

**Published:** 2025-12-17

**Authors:** Mark I. M. Noble, Angela J. Drake‐Holland

**Affiliations:** ^1^ Royal Society of Medicine London UK

## 1 INTRODUCTION

We have considered the published material on the subject of control of the heart rate that depends upon sino‐atrial pacemaker function, and consider that an explanation based on the gain of an unknown cation, the ‘funny current’ (*I*_f_), is counter‐intuitive. The positivity of the diastolic drift is more simply explained by loss of negative charge from its maximum at the end of re‐polarisation (end of previous action potential). There also does not seem to be clarity on the mechanism of the threshold for action potential generation. We suggest that it is due to release of immobilised ions as the electric field force declines to an insufficient force for its maintenance.

We propose that in sino‐atrial pacemaker cells, the change in diastolic electric potential is due to loss of electric negatively charged particles, not an unknown positively charged ion current (‘funny current’, *I*_f_). In sino‐atrial pacemaker cells in diastole there is an electric field force (transmembrane potential divided by membrane thickness) that imobilises ions and declines in diastole with the decline in transmembrane potential. The threshold for action potential formation occurs when the electric field force is no longer strong enough to maintain ionic immobilisation.

This frees calcium (Ca^2+^) and sodium (Na^+^) to form action potentials and to participate in intracellular events. In the case of potassium ions (K^+^) the predominant effect is exclusion from the cell into the extracellular compartment along its concentration gradient; presumably the K^+^ is acquired by Na^+^ pumping and its removal maintains the K^+^ steady state.

## 2 EFFECTS ON HEART RATE

Heart rate (pulse rate) in normal mammals is set by the interval between action potentials (AP) of the sino‐atrial pacemaker cells of the right atrium. This frequency passes through the conduction system of the heart (atria to AV node to His bundle fibres to bundle branches (Purkinje cells) to ventricular muscle).

The subsequent transition to an action potential is at the ‘threshold’ potential. Our study aims to deduce a possible hypothesis to explain the mechanism of this transition.

**FIGURE 1:**
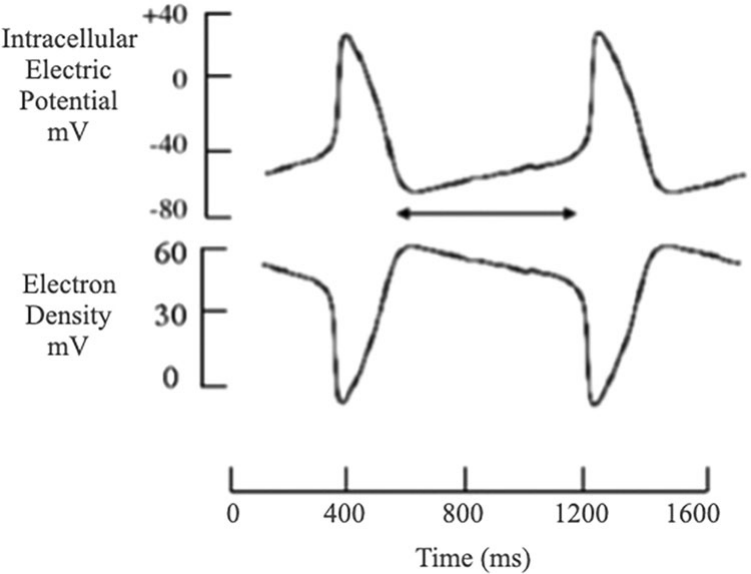
Sino‐atrial node cell recording. Top trace is an example of spontaneous electrical events in a sino‐atrial pacemaker cell. Bottom trace shows corresponding events at electron level, calculated from top trace according to √[*V*]2. This suggests that the diastolic downward drift of the transmembrane potential during the double arrow is caused by loss of or leakage of electrons through an electrical resistance.

The determinants of the sino‐atrial AP and heart rate frequency (Figure 1) are therefore important to understand fully, but this is only partially the case at present (Lakatta & DiFrancesco, 2009) and is controversial (see references). The factors most prominent in the literature are the ‘funny current’ (*I*_f_) in diastole, and the role of calcium ions (Ca^2+^) during the action potentials.

A purpose of the present communication is to draw attention to the neglected factor of intracellular electricity defined by Systeme Internationale as electrons moving (Noble, 2023). In diastole, when the cell membrane is not interrupted by APs, the transmembrane potentials of the individual cell form a continuous electrical field (Taghian et al., 2012; Veech et al., 1995).

## 3 HOW DOES THIS HELP US UNDERSTAND THE MECHANISM OF THE THRESHOLD FOR AP PRODUCTION?

The determinants of the sino‐atrial AP and heart rate frequency (Figure 1) are therefore important to understand fully, but this is only partially the case at present and is controversial (Lakatta & DiFrancesco, 2009, Lakatta & Maltsev, 2012). The factors most prominent in the literature are the ‘funny current’ (*I*_f_) in diastole, and the role of calcium ions (Ca^2+^) during the action potentials. The purpose of the present communication is to draw attention to the neglected factor of intracellular electricity (defined by Systeme Internationale as electrons moving [Noble, 2023. In diastole, when the cell membrane is not interrupted by APs, the transmembrane potentials of the individual cell form a continuous electrical field [Taghian, Narrmoneva & Kogani, 2012, Veech, Kashiwayaa & King, 1995]. How does this help us understand the mechanism of the threshold for AP production?

## 4 DEVELOPMENT OF A HYPOTHESIS

### 4.1 The effect of electric field force on the ions within the cell

The electric field is very high in diastole and is affected by a force that is independent of its velocity but dependent on its frequency. The electric potential difference across the membrane of a resting cell is around −70 mV, the membrane's thickness is 5 nm, and the corresponding electric field strength is about 107 V/m inside the membrane leaflets (Taghian et al., 2012). It follows that this enormous force applies in diastole (Figure 1) between transmembrane potentials of approximately −60 mV to approximately −40 mV, at which the threshold mechanism occurs, after which the AP rapidly takes the transmembrane potential and electric field force to zero.

### 4.2 Why and how?

*Why?* The mechanism – whatever it is – has presumably evolved because it conveys an advantage to the organism.

**FIGURE 2:**
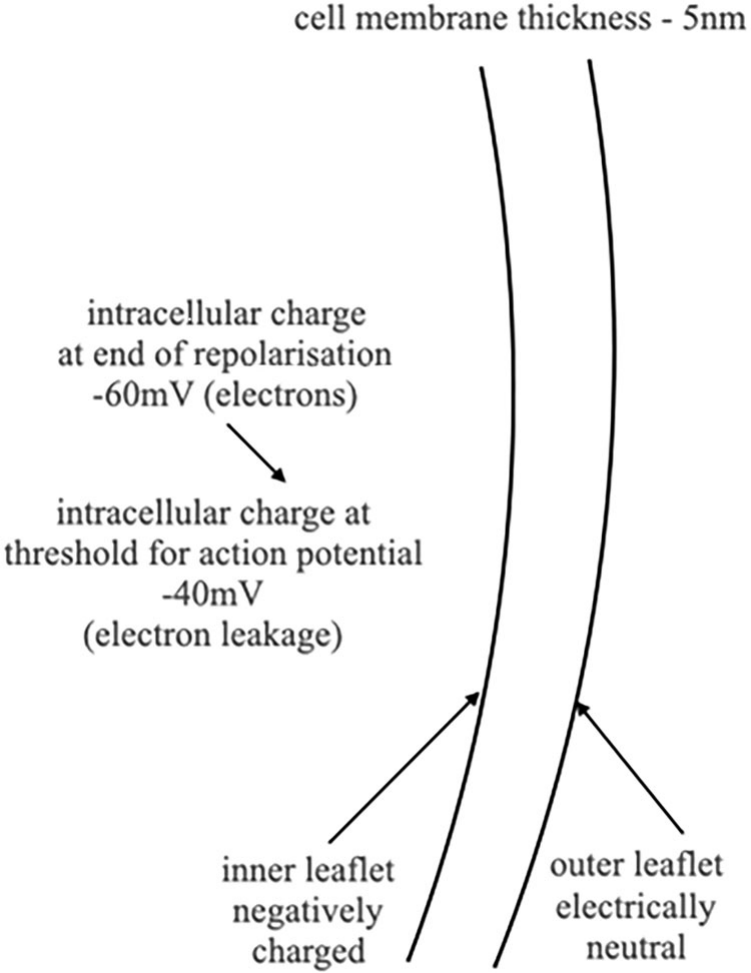
Diagram of new concept. The drift in electrical potential during diastole (Figure 1) is postulated to be a gradual loss of electrons, bringing the intracellular potential from −60 mV down to −40 mV. 40mV is the trigger value for initiation of the next following action potential and consequent heartbeat.

*How?* What is happening to Ca^2+^ during the threshold transformation? There was much past evidence of diastolic Ca^2+^ binding to the inner sarcoplasmic leaflet in diastole by Langer (1985, 2012, 1986), and Borgers et al. (1985, 1984), which is consistent with the concept of ionic immobilisation (of Ca^2+^) by the electric field. The strength of the electric field declines slowly with the decline in transmembrane potential (Figure 2). In the perfusate when studying muscle cells with the cell membrane removed, an extremely low Ca^2+^ concentration (Fabiato & Fabiato, 1975; Kentish et al., 1986) is necessary to prevent cell death.

We prefer to think that the diastolic decline of transmembrane potential, and therefore of electric field strength, is due to loss of the negative charges of the electrons, rather than gain of positive electric charge (*I*_f_, of unknown particle movement).

We postulate that weakening of electric field force allows ‘freedom’ for Ca^2+^ in the sarcolemma and the other formerly immobilised ions to flow out from their former ‘prisons’ to produce the action potential. The Ca^2+^ is also free to flow inwards to the cytoplasm where it can participate in intracellular calcium ion handling to allow contraction, and sodium/calcium exchange (NCX, see Kronhaus et al., 1978).

Consistent with this idea is the simultaneous release of formerly restrained potassium ions into the extracellular fluid (Kronhaus et al., et al., 1978).

Repolarisation after the action potential is equally important to depolarisation in order to allow intermittency of the system. We postulate that restoration of diastolic electricity could be achieved by mitochondrial supply (Noble, 2023).

Changes in the interval between APs and heart rate could be achieved if the causative agent has an end‐effect on the electrical resistance, for example, catecholamines reduce resistance to shorten the interval between APs; acetylcholine increases resistance to lengthen AP interval duration.

## 5 CONCLUSION AND HYPOTHESIS

The occurrence of a ‘threshold’ for action potentials in sino‐atrial cells could be attributed to the diastolic decline in electric field strength to a value that is unable to maintain the electric restraint on cation movement.

## AUTHOR CONTRIBUTIONS

Both authors have read and approved the final version of this manuscript and agree to be accountable for all aspects of the work in ensuring that questions related to the accuracy or integrity of any part of the work are appropriately investigated and resolved. All persons designated as authors qualify for authorship, and all those who qualify for authorship are listed.

## CONFLICT OF INTEREST

There are no conflicts of interest.

## FUNDING INFORMATION

No funding was received for this work.
